# Hospitalization at the end of life among nursing home residents with dementia: a systematic review

**DOI:** 10.1186/s12904-019-0462-1

**Published:** 2019-09-10

**Authors:** Falk Hoffmann, Anke Strautmann, Katharina Allers

**Affiliations:** Department of Health Services Research, Carl von Ossietzky University Oldenburg, Ammerländer Heerstr. 140, 26129 Oldenburg, Germany

**Keywords:** Nursing homes, Death, End-of-life care, Long-term care, Hospital use, Health services research

## Abstract

**Background:**

Half of nursing home residents (NHR) suffer from dementia. End-of-life hospitalizations are often burdensome in residents with dementia. A systematic review was conducted to study the occurrence of hospitalizations at the end of life in NHR with dementia and to compare these figures to NHR without dementia.

**Methods:**

A systematic literature search in MEDLINE, CINAHL and Scopus was conducted in May 2018. Studies were included if they reported proportions of in-hospital deaths or hospitalizations of NHR with dementia in the last month of life. Two authors independently selected studies, extracted data, and assessed quality of studies.

**Results:**

Nine hundred forty-five citations were retrieved; 13 studies were included. Overall, 7 studies reported data on in-hospital death with proportions ranging between 0% in Canada and 53.3% in the UK. Studies reporting on the last 30 days of life (*n* = 8) varied between 8.0% in the Netherlands and 51.3% in Germany. Two studies each assessed the influence of age and sex. There seem to be fewer end-of-life hospitalizations in older age groups. The influence of sex is inconclusive. All but one study found that at the end of life residents with dementia were hospitalized less often than those without (*n* = 6).

**Conclusions:**

We found large variations in end-of-life hospitalizations of NHR with dementia, probably being explained by differences between countries. The influence of sex and age might differ when compared to residents without dementia. More studies should compare NHR with dementia to those without and assess the influence of sex and age.

**Trial registration:**

PROSPERO registration number CRD42018104263.

## Introduction

Dementia is one of the most important reasons for transitions to nursing homes in elderly people and the prevalence of dementia in nursing home residents (NHR) is much higher compared to community-dwelling older adults [1–3]. Although there are variations in the literature, most studies found that about 50% or even more of NHR suffer from dementia [4–9]. Residents with dementia differ in many important aspects from those without. They are typically older [7, 10], need more support to manage activities of daily living and behavioral problems [10, 11] and spend, on average, a longer time in the nursing home before death compared to residents without dementia [12, 13]. Due to the irreversible and slowly progressive nature of the disease, those affected usually become more and more unable to participate in decisions about medical care [14] and often die from complications of dementia [15, 16]. This suggests that end-of-life care might also differ from residents dying from other diseases.

Hospitalizations at the end of life do not only lead to a substantial economic burden, but they are often not beneficial for NHR [17]. Some studies even define in-hospital deaths of NHR occurring within 3 days of admission to be burdensome or inappropriate [18, 19]. There seems to be a large variation in the literature on hospitalizations of NHR with dementia at the end of life, even in bordering countries. For instance, a study from the Netherlands found that 8.0% of residents with dementia were hospitalized in the last month of life [20], whereas Belgian data revealed 19.5% [21] and a recent German study even showed a much higher proportion of 51.3% [13]. Furthermore, the literature is inconclusive on whether hospitalizations at the end of life differ between NHR with and without dementia. There are studies showing a much lower proportion of hospitalizations at the end of life in NHR with dementia compared to those without [15, 22], other analyses found no differences [13]. Although the evidence seems to be largely inconclusive, to our knowledge, no systematic review on these questions has been done yet.

Therefore, our aim was to give an overview on the existing literature on a) the occurrence of hospitalizations at the end of life in NHR with dementia and b) to compare these figures to NHR without dementia in the subset of studies reporting both groups.

## Methods

A protocol for this systematic review was registered with PROSPERO (CRD42018104263). We followed the Preferred Reporting Items for Systematic Reviews and Meta-Analysis (PRISMA) statement for reporting [23].

### Data sources and search

The literature search was performed with the databases MEDLINE (via PubMed), CINAHL and Scopus. The search strategies for dementia [24] and end-of-life hospitalization of NHR were adapted from prior systematic reviews [25, 26] (see Additional file 1: Table S1 for search strategy). We searched the electronic databases from inception to 14 May 2018. Additionally, we scanned the reference lists of all included studies.

### Eligibility criteria

We defined study eligibility criteria using the CoCoPop (condition, context, and population) approach for reviews assessing prevalence and incidence data [27, 28].

#### Condition

We included studies reporting on proportions of all-cause hospitalizations occurring during any defined period in the last month of life (e.g. the last 30 days, 14 days or 7 days or in-hospital deaths, as reported by the authors). If a study only reported hospitalization due to specific diagnoses it was excluded.

#### Context

As previous research, we included studies of nursing homes, care homes, long-term care, skilled nursing or residential care facilities [25, 26]. Studies reporting on participants from other forms of care were only included if they contained specific data about NHR. We excluded studies on assisted living facilities or long-term care hospitals. Studies containing nursing homes with specific characteristics (e.g. veteran nursing homes, specific religious tendencies) were not excluded.

#### Population

The studies had to contain data on deceased NHR with dementia. NHR were considered to have dementia if the authors labelled participants to have dementia or if they used some form of cognitive impairment scale and gave a cut-off for dementia. If participants were only labelled as having cognitive impairment without being classified as having dementia, the study was excluded. Studies limited to specific groups of residents (i.e. specific diagnoses other than dementia) were also excluded.

Published observational and interventional studies were included. We excluded interventional studies without control groups or not reporting baseline data, PhD theses, and studies with a sample size smaller than 20 deceased residents. No other limitations, including language and location of publication, were applied.

### Study selection and data extraction

After exporting citations into an EndNote library and removing duplicates, two of the authors independently screened articles based on title and abstract for inclusion or exclusion. Full texts of all articles that met the inclusion criteria were independently assessed by the two reviewers and any disagreement was resolved by discussion or by a third reviewer.

We abstracted data on study characteristics (e.g., country, data source, assessment of dementia), resident characteristics (e.g., mean age, sex) and outcome results using a standardized data abstraction form. Data extraction was performed by one reviewer and verified by a second. Discrepancies were resolved by discussion or by a third reviewer.

When the proportion of residents with end-of-life hospitalizations was not directly specified in the publication, we calculated it, whenever possible, by dividing the number of deceased NHR with dementia hospitalized by the total number of deceased residents with dementia. If the original publication stratified its results by different groups, such as race or location of the nursing home, we reported the total proportion of hospitalizations for all NHR with dementia. When a study reported proportions for several years, only the latest year or period was included.

### Quality assessment

The quality of the included studies was assessed using the Joanna Briggs Institute’s (JBI) critical appraisal checklist for studies reporting prevalence data, which includes nine items [27]. We chose this tool because of its flexibility across different study designs [29]. Two reviewers independently appraised the quality of included studies. Any disagreement was resolved by discussion. If necessary a third reviewer was involved. Study quality had no impact on the inclusion or exclusion of studies.

### Data synthesis

We analysed the results using a narrative synthesis. Due to the expected heterogeneity between studies, a meta-analysis was not planned.

Differences in hospitalization regarding age and sex were analysed as far as they were reported (irrespective of whether stratified proportions were presented or if these variables were included in regression models). Additionally, differences in end-of-life hospitalizations between NHR with dementia and those without dementia were analysed in studies which compared both groups.

We initially also planned to assess differences by severity of dementia, but refrained from this due to the very heterogeneous ways of assessing dementia.

## Results

### Literature search

**Fig. 1:**
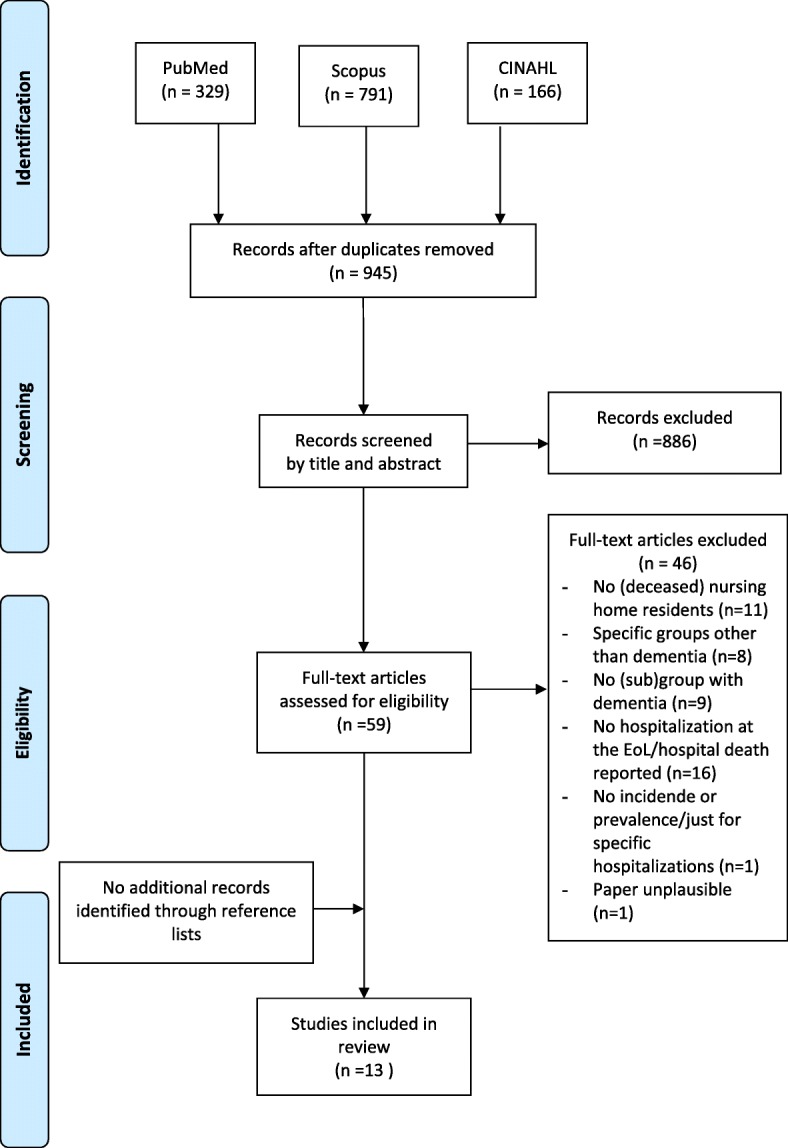
Flowchart of the literature search

After screening 945 titles and abstracts and 59 full text articles, 13 studies met the inclusion criteria (Fig. 1) [13, 15, 20–22, 30–37]. All were reported in English. No additional studies were identified through screening of reference lists.

### Study and patient characteristics

The 13 studies were published between 2005 and 2018, with 10 studies being published from 2013 onwards (76.9%). All studies were conducted in western industrialised countries; 6 in the USA (46.2%), and one each in Finland, Australia, Germany, the Netherlands, Belgium, Canada and the UK (Table 1). The sample size ranged from 30 to 1,261,726 deceased NHR with dementia.

**Table 1:** Baseline characteristics of the studies included

First author, year	Country	Data source	Year of data	Sample (with dementia)	Inclusion/ exclusion criteria	How was dementia assessed?	Mean age at death (% females)
Aaltonen, 2014 [33]	Finland	Nationwide registry data (Care register for health care and Care register for social care) Causes of Death Register	2002–2008	13,159^a^ NHR	Died at 70 years or older In NH during their last months of life (in care both 6 months and 3 months before death, stayed there for ≥90 days during the last 6 months of life)	Any cause of death (immediate, underlying, intermediate, contributing) with ICD-10 codes: F00, F01, F02, F03, G30	87 years (76%)
Agar, 2017 [34]	Australia	Face-to-face or telephone interviews Nursing home and medical records Questionnaires	2013–2014	64^b^ NHR from 10 NH	Nursing homes with ≥50% of residents with dementia providing intensive levels of care Residents with advanced dementia	Medical records, FAST, AKPS Advanced dementia: - documented diagnosis of dementia - and FAST (≥6a, stable for 1 month) - and AKPS ≤50	85.8 years (58%)
Allers, 2018 [13]	Germany	Health insurance claims data (DAK) Long-term care insurance data	2010–2014	not reported	≥65 years old, newly admitted to a NH Insured continuously for at least 365 days without NH placement before	ICD-10 codes in the quarter of NH admission: F00.x, F01.x, F02.0, F02.3, F03, G30.x, G31.0, G31.1, G31.82, G31.9, R54	not reported
Cai, 2016 [35]	USA	MDS 2.0 Medicare beneficiary summary file Medicare claims	2007–2010	293,967 NHR	≥65 years old In NH ≥90 days before death Continuously enrolled in Medicare fee-for-service Plans Medicare–Medicaid dually eligible during the last 30 days of life	CPS constructed of MDS 2.0 data - mild cognitive impairment: CPS 0–2 - moderate cognitive impairment: CPS 3–4 - severe cognitive impairment: CPS 5–6	CPS 3–4: 85.9 years (70.1%)^c^ CPS 5–6: 85.7 years (76.3%)^c^
Gessert, 2008 [36]	USA	Administrative databases from Centers for Medicare and Medicaid services MDS	2000–2001	3703 NHR	Urban and rural NH ≥67 years old Not enrolled in Health Maintenance Organization No hospice benefits during 2 years prior to death Not comatose	Severe and persistent cognitive impairment based on the CPS - CPS = 6 on ≥2 consecutive MDS reports at least 60 days apart - absence of MDS reports with CPS ≤4 there after	87.1 years (76.4%)^c^
Hendriks, 2017 [20]	Netherlands	Questionnaires from the Dutch end-of-life in dementia study (DEOLD)	2007–2011	330 residents from 34 LTCF	Residents admitted to psychogeriatric wards	Known diagnosis of dementia upon nursing home admission (by a physician) Advanced dementia: CPS 5–6 + GDS 7	85.2 years (not reported)
Houttekier, 2014 [21]	Belgium	Questionnaires (with access to medical files) from the “Dying well with Dementia” study	2010	195 NHR from 69 NH	Inclusion process in two steps: First: residents had to be completely care dependent for ADL and disoriented in time and space or had to have a Katz scale score ≥ 3 Second: Resident had to have dementia (reported by nurse or GP)	After inclusion CPS and GDS was assessed: - mild/moderate: CPS < 5, GDS < 7 - severe: CPS ≥5 and GDS < 7 or CPS < 5 and GDS = 7 -very severe/ advanced: CPS ≥5, GDS = 7	< 85 years: 31.7% 85–90 years: 37.7% > 90 years: 30.6% (61.4%)
Krishnan, 2015 [37]	Canada	MDS 2.0 Medical charts Death certificates	2010–2013	58 NHR from 1 NH	All residents were included	Dementia recorded on death certificate as underlying or immediate cause of death	not reported
Lamberg, 2005 [30]	USA	MDS Medical long-term care records	2001–2003	240 NHR from 1 NH	Long term residents (stay ≥30 days) Advanced dementia (CPS 5–6)	CPS 5–6 Long term care medical records to identify cause of cognitive impairment	92 years (median) (75.8%)
Li, 2013 [22]	USA	MDS 2.0 Medicare beneficiary file Hospice and hospital claims	2003–2007	143,980 NHR^c^	≥65 years old > 3 months in nursing home No rehabilitation or postacute stay Not comatose No transfers to another NH after last assessment Not enrolled in managed care in last 30 days of life	Diagnosis of Alzheimer’s disease or other dementia on the last full MDS assessment	87.4 years (71.9%)^c^
Livingston, 2013 [31]	UK	Care home resident records Interviews Questionnaires		30 NHR^d^ from 1 NH	Living in the NH for at least 1 month before death Resident records had to be available	Medical records Diagnosis of dementia or suspected dementia (symptoms fulfilling standard dementia criteria)	not reported
Sloane, 2008 [15]	USA	(Telephone) interviews with staff and family Study cohort from the Collaborative Studies of long-term care (CS-LTC)	2002–2005	247 NHR	NHR who spent 15 out of 30 days in a NH Died no more than 3 days after leaving the NH	NH staff members were asked whether the decedent was an Alzheimer’s type resident 3 months before death and if dementia was a contributing factor towards the resident’s death	not reported
Temkin-Greener, 2013 [32]	USA	Nationwide administrative data from the chronic condition data warehouse Medicare denominator files MDS	2003–2007	384,355 NHR	Died in NH or within 8 days of discharge to a different care setting Not enrolled in managed care in last 30 days of life Not from Virgin Islands or Puerto Rico	not reported	not reported

Proportions are reported with one decimal place (provided decimal places were given or could be calculated)

*Abbreviations*: *ADL* Activities of daily living, *AKPS* Australia-modified Karnofsky Performance Status, *CPS* Cognitive Performance Scale ranging between 0 (intact cognitive performance) and 6 (very severely impaired), *FAST* Functional Assessment Staging Tool, *GDS* Global Deterioration Scale ranging from 1 (no cognitive decline) to 7 (very severe cognitive decline), *LTCF* Long-term care facility, *MDS* Minimum Data Set, *NH* Nursing home, *NHR* Nursing home residents

^a^Sample size given in this table does not represent the whole study population but only the number of nursing home residents reported

^b^Study design was an intervention study with a control group, the data reported in this table refers to the control group

^c^Numbers refer to the latest year studied, which was 2007

^d^Numbers refer to residents who died prior to the intervention

Overall, 8 studies each reported data on age and sex of deceased NHR with dementia. Mean or median age varied between 85 and 92 years. One study reported age categories (< 85 years: 31.7%; 85–90 years: 37.7%; > 90 years: 30.6%). The proportion of females ranged between 58 and 76.4%.

Eleven studies used retrospective design. Six studies used some form of medical records such as Minimum Data Set (MDS) or care home records. Dementia was assessed in a variety of ways with studies obtaining diagnoses from claims data, registries, medical records or interviews.

### Methodological quality of included studies

The quality assessment for each study is shown in Table 2. In 9 studies (69.2%) the sample frame was appropriate to address the target population. In two of the studies (15.4%) both dementia and end-of-life hospitalization were assessed with valid methods, six studies did not use valid methods and in five studies it was unclear whether valid methods were used or not.

**Table 2:** Summary of quality assessment

First Author, Year	1	2	3	4	5	6	7	8	9
Aaltonen, 2014 [33]	Yes	Yes	Yes	No	N/A	No	Yes	No	N/A
Agar, 2017 [34]	No	Yes	No	No	Yes	Unclear	Unclear	Yes	Yes
Allers, 2018 [13]	Yes	Yes	Yes	No	N/A	No	Yes	No	N/A
Cai, 2016 [35]	Yes	Yes	Yes	Yes	N/A	Unclear	Yes	Yes	N/A
Gessert, 2008 [36]	Yes	Yes	Yes	No	N/A	Yes	Yes	Yes	N/A
Hendriks, 2017 [20]	Yes	Yes	No	No	Yes	Unclear	Unclear	Yes	Yes
Houttekier,2014 [21]	Yes	Unclear	No	Yes	Yes	Unclear	No	Yes	Yes
Krishnan, 2015 [37]	No	Yes	No	No	Yes	No	Yes	Yes	N/A
Lamberg, 2005 [30]	No	Yes	No	Yes	Unclear	Yes	Unclear	No	N/A
Li, 2013 [22]	Yes	Yes	Yes	Yes	N/A	No	Yes	Yes	N/A
Livingston, 2013 [31]	No	Yes	No	No	Unclear	No	Unclear	Yes	Unclear
Sloane, 2008 [15]	Yes	Yes	No	Yes	Unclear	No	Unclear	Yes	Yes
Temkin-Greener, 2013 [32]	Yes	Yes	Yes	No	N/A	Unclear	Yes	Yes	N/A

Quality appraisal criteria [27]:

1) Was the sample frame appropriate to address the target population?

2) Were the study participants sampled in an appropriate way?

3) Was the sample size adequate?

4) Were the study subjects and the setting described in detail?

5) Was the data analysis conducted with sufficient coverage of the identified sample?

6) Were valid methods used for the identification of the condition?

7) Was the condition measured in a standard, reliable way for all participants?

8) Was there appropriate statistical analysis?

9) Was the response rate adequate, and if not, was the low response rate managed appropriately?

*Abbreviations*: *N/A* Not applicable

### In-hospital deaths

Overall, 7 of the included studies reported data on in-hospital death of NHR with dementia [15, 22, 30–33, 37], with proportions ranging between 0% in Canada and 53.3% in the UK (Table 3). The 4 studies from the USA showed in-hospital deaths from 4.2 to 15.1%.

**Table 3:** Results of the studies included

First author, year	Country (sample size)	In-hospital death	Other period before death
Aaltonen, 2014 [33]	Finland (*n* = 13,159)	20.6%^a^	
Agar, 2017 [34]	Australia (*n* = 64)		30 days: 18%^b^
Allers, 2018 [13]	Germany (not reported)		7 days: 36.8% 30 days: 51.3%
Cai, 2016 [35]	USA (CPS 3–4: *n* = 189,219, CPS 5–6: *n* = 104,748)		30 days CPS 3–6: 29.6%^a^ CPS 3–4: 32.5%^a^ CPS 5–6: 24.3%^a^
Gessert, 2008 [36]	USA (*n* = 3703)		30 days: 32.4%
Hendriks, 2017 [20]	Netherlands (*n* = 330)		7 days: 1.5% 30 days: 8.0%
Houttekier, 2014 [21]	Belgium (*n* = 195)		30 days: 19.5%
Krishnan, 2015 [37]	Canada (*n* = 58)	0%	
Lamberg, 2005 [30]	USA (*n* = 240)	4.2%	30 days: 8.3%
Li, 2013 [22]	USA (*n* = 143,980)^c^	14.2%^c^	
Livingston, 2013 [31]	UK (*n* = 30)	53.3%^d^	
Sloane, 2008 [15]	USA (*n* = 247)	6.9%	30 days: 23.6%
Temkin-Greener, 2013 [32]	USA (*n* = 384,355)	14.4%	

^a^Calculated from data given in the publication

^b^Calculations presented here are taken from the original publication but were not reproducible

^c^Numbers refer to the latest year studied, which was 2007

^d^Study design was Intervention study. Data given here does only refer to the residents of the control group

None of these studies analysed differences regarding age or sex.

### End-of-life hospitalization during other periods

Overall, 8 studies reported data on end-of-life hospitalizations for other periods during the last month of life [13, 15, 20, 21, 30, 34–36]. Of them, 2 studies reported on the last 7 days and 8 studies on the last 30 days of life. The amount of hospitalization during the last 30 days of life varied substantially between 8.0% in the Netherlands and 51.3% in Germany. Besides this German study, the proportion of NHR being hospitalized was up to 32.4% in the remaining studies.

Those 2 studies with the lowest and highest amount of NHR hospitalized during the last month of life also reported on the last 7 days before death. The Dutch study found that 1.5% were hospitalized and the German one reported 36.8%.

Two of the 8 studies also analysed differences with respect to age or sex and both assessed the last 30 days of life. Houttekier et al. reported stratified proportions and found that 20.4% of female and 18.3% of male residents experienced end-of-life hospitalization [21]. Among decedents with dementia aged less than 85 years, 22.4% were hospitalized during the last month of life compared to 18.8% in those aged between 85 and 90 years and 16.1% in residents older than 90 years. Cai et al. reported results from a multivariate logistic regression analysis [35]. They found that males with moderate as well as severe cognitive impairment were slightly more likely to experience end-of-life hospitalization. Older age was associated with fewer end-of-life hospitalizations within both the moderate and severe cognitive impairment groups.

### Differences between decedents with and without dementia

This review includes 6 studies which compare end-of-life hospitalization of deceased NHR with and without dementia [13, 15, 22, 32, 35, 37]. The studies are from the USA (*n* = 4) and one study each from Canada and Germany. Only 2 studies reported baseline data stratified for both groups and found that decedents with dementia were older. With the exception of one, all other studies found that residents with dementia experience less hospitalization than those not suffering from dementia (Table 4).

**Table 4:** Results of the studies comparing residents with dementia and without dementia

First author, Year	Residents with dementia		Residents without dementia	
	Mean age at death (% females, sample size)	Hospitalization before death	Mean age at death (% females, sample size)	Hospitalization before death
Allers, 2018 [13]	not reported	7 days: 36.8% 30 days: 51.3%	not reported	7 days: 37.8% 30 days: 51.6%
Cai, 2016 [35]	CPS 3–4: 85.9 (70.1%, *n* = 189,219) CPS 5–6: 85.7 (76.3%, *n* = 104,748)	30 days: CPS 3–6: 29.6%^a^ CPS 3–4: 32.5%^a^ CPS 5–6: 24.3%^a^	CPS 0–2: 84.0 (69.5%, *n* = 100,981)	30 days: 42.8%
Krishnan, 2015 [37]	not reported (*n* = 58)	In-hospital death: 0%	not reported (*n* = 60)	In-hospital death: 11.7%^a^
Li, 2013 [22]	87.4 (71.9%, *n* = 143,980)	In-hospital death: 14.2%	85.2 (66.9%, *n* = 92,639)	In-hospital death: 19.7%
Sloane, 2008 [15]	not reported (*n* = 247)	In-hospital death: 6.9% 30 days: 23.6%	not reported (*n* = 67)	In-hospital death: 13.8%^b^ 30 days: 34.3%
Temkin-Greener, 2013 [32]	not reported (*n* = 384,355)	In-hospital death: 14.4%	not reported (*n* = 1,845,014)	In-hospital death: 20.7%

^a^Calculated from data given in the publication

^b^Calculations presented here are taken from the original publication but were not reproducible

Four of the six studies which compared NHR with and without dementia reported their in-hospital deaths. Krishnan et al. found that none (0%) of the Canadian residents with dementia died in hospital compared to 11.7% of those without [37]. Some smaller differences for in-hospital deaths were also found in the studies by Sloane et al. (6.9% with dementia vs. 13.8% without) [15], Li et al. (14.2% vs. 19.7%) [22] and Temkin-Greener et al. (14.4% vs. 20.7%) [32], all were conducted in the USA.

Three of the six studies which compared NHR with and without dementia reported their hospitalizations during the last 7 or 30 days of life. Two studies conducted in the USA reported proportions of 23.6% vs. 34.3% [15] and 29.6% vs. 42.8% [35] respectively for hospitalizations of NHR with and without dementia in the last month of life. The study by Cai et al. also reported a clear trend with 24.3, 32.5 and 42.8% in residents with severe, moderate and no or mild cognitive impairment [35]. The only study showing no difference between residents with and without dementia for hospitalizations during the last 7 (36.8% vs. 37.8%) and 30 days of life (51.3% vs. 51.6%) was the German one [13].

## Discussion

### Comparison with other studies and interpretation

In this systematic review, we found large variations in end-of-life hospitalizations of NHR with dementia, probably being explained by differences between countries. Most studies were from the USA. Only two studies assessed the influence of age or sex. There seems to be a trend towards fewer end-of-life hospitalizations in older age groups, but the influence of sex is inconclusive. All but one study found that at the end of life residents with dementia were hospitalized less often than those without.

The proportion of in-hospital deaths and end-of-life hospitalizations ranged widely from 0 to 53% and 8–51%, respectively. This is in line with our previous systematic review on end-of-life hospitalization of all NHR which also showed large variations between the included studies [26]. These differences might partly be explained by the different health care systems and long-term care structures as well as differences in qualifications and attitudes regarding end-of-life care across countries [38, 39]. Also, another study which focused on place of death in all people with dementia found that nursing home and in-hospital deaths differed significantly between five European countries with a decreased chance of nursing home death in regions with more hospital beds [39]. But also within-country variations resulting, for example, from different availability of healthcare resources, regional policy regulations or local cultures might play a role [32, 40, 41].

Most studies found that residents with dementia were less often hospitalized at the end of life which indicates a less aggressive treatment among individuals with dementia. Only the study from Germany found no difference between residents with and without dementia [13]. One explanation for this might be that palliative care is more common in other countries compared to Germany. This is supported by the fact that knowledge on palliative care is low among staff in German nursing homes [42]. This could also explain the higher overall rate of end-of-life hospitalization in Germany.

Hospitalizations at the end of life are often burdensome and potentially avoidable, especially in NHR with dementia [19, 43]. Some studies reported very low proportions of end-of-life hospitalization in NHR with dementia while others did not. This might be due to differences in the dissemination of palliative care. Palliative care provision is associated with a decrease in end-of-life hospitalization [20, 21, 44]. Therefore, early communication with residents and their relatives about palliative care approaches and treatment preferences is important to enhance quality of life and to improve end-of-life care [20].

We found only 2 studies assessing the influence of age on end-of-life hospitalizations in NHR with dementia [21, 35]. Both reported that older residents were hospitalized less often than younger ones. However, just one of them stratified their analysis by 3 different age groups and showed a clear linear trend. The other study included age as a linear variable in the regression. At first sight, these results are quite comparable to the literature on hospitalizations during end of life of all NHR, not just restricting to those with dementia. In our recent systematic review of 38 studies, most but not all of the 15 studies investigating the influence of age found that younger age was associated with a higher probability of end-of-life hospitalizations in all NHR [26]. A more recent in-depth analysis of the German study included in this review [13] was the first research comparing proportions of in-hospital death between NHR with and without dementia by age. Using 4 age groups, this study showed a clear linear decrease from 37.0 to 20.2% when comparing NHR with dementia aged 65–74 and 95+ years. Interestingly, in residents without dementia in-hospital deaths showed an inverse U-shaped distribution (24.6, 32.0, 30.9 and 22.9% for age groups 65–74, 75–84, 85–94 and 95+ years). [45] Moreover, a systematic review on all-cause hospitalizations of NHR residents also showed less consistent findings regarding age suggesting that its influence is not linear [25]. Taken together, these results highlight less aggressive treatment approaches towards death with increasing age especially in NHR with dementia. This might be explained by the fact that fewer benefits are expected from hospitalizations in this population, especially in older age. However, decision-making about whether a resident with or without dementia will benefit from a hospital admission or not is sometimes challenging and it is often difficult to know when a resident is near the end of life [46].

Surprisingly, the influence of sex on end-of-life hospitalizations of NHR with dementia was quite inconclusive. In our recent systematic review on end-of-life hospitalizations of all NHR, some but not all studies indicated that male sex was associated with a higher probability of hospitalization [26]. However, in our systematic review on overall hospitalizations of NHR, this finding was consistent: all 20 studies assessing the influence of sex found that hospitalisations occur more often in male NHR [25]. This might suggest that sex has a smaller influence on hospitalizations during end-of-life than in periods before, especially in NHR with dementia. However, one has to keep in mind that only 2 studies included in this review assessed the effect of sex [21, 35] and one of them only had a sample size of 195 decedents [21].

Taken together, there is a clear need for further studies which compare end-of-life hospitalizations and possible reasons for differences between NHR with and without dementia. These studies should have large sample sizes that allow comparing predictors of end-of-life hospitalizations between these very different groups. Especially age and sex should also be considered in future studies on end-of-life hospitalization in NHR with dementia.

### Strengths and limitations

The main strength of this review was its broad search without language restrictions. However, some of the included studies did not explicitly focus on end-of-life hospitalization or in-hospital death of NHR with dementia as their main research question but also reported some data briefly in the full text. Therefore, we might have missed studies which seemed not to be eligible based on their title and abstracts. However, we screened about 60 abstracts and also searched references lists of included studies in order to minimise the risk of missing studies. It has to be taken into account that, besides the primary research question, the included studies are of great heterogeneity regarding study design, sample size, sample characteristics and the way dementia was assessed. Furthermore, it is often not clear whether studies reporting on other measures than in-hospital death assess being in hospital or just new admissions to hospital during the respective period. This heterogeneity has to be considered when comparing the results of the different studies. However, we assessed the quality of each study according to the JBI tool recommended for systematic reviews of studies on prevalence [27] and explained the results of the quality appraisal transparently in this review. For some items quality was rather low, e.g., in the majority of studies both dementia and end-of-life hospitalization were not assessed with valid methods which has to be taken into account when interpreting the results.

### Conclusions and implications

We found a large variation of end-of-life hospitalization of NHR with dementia, which seems to be explained to a large extent by the country in which the study was conducted. However, more studies from outside the USA and from countries other than western industrialised are needed. Only 2 studies assessed the influence of age showing that older decedents seem to be hospitalized less often. The 2 studies on the influence of sex show no clear picture. Most studies found that NHR with dementia were hospitalized less often at the end of life compared with those without. The influence of sex and age on end-of-life hospitalization might also differ from those in all residents. However, more studies comparing NHR with dementia to those without and assessing the influence of sex and age are needed. Given the burden end-of-life hospitalization can cause for residents with dementia and the large differences between countries, it would be highly desirable to better understand best practices and structures of healthcare systems in which low proportions of such hospitalizations occur in order to improve end-of-life care worldwide.

## Supplementary information

**Additional file 1: Table S1.** Search strategy.

## Data Availability

Not applicable

## Abbreviations

JBI: Joanna Briggs Institute
NHR: Nursing home residents
PRISMA: Preferred Reporting Items for Systematic Reviews and Meta-Analysis
