# Supplementary material for: Hospitalization at the end of life among nursing home residents with dementia: a systematic review
Source: BMC Palliat Care. 2019 Sep 10;18:77. doi: 10.1186/s12904-019-0462-1 (PMC6737675; doi:10.1186/s12904-019-0462-1)
Supplement: Supplementary file 1 — Additional file 1: Table S1. Search strategy. [file 12904_2019_462_MOESM1_ESM.docx]

Appendix

Table 1.Search strategy

| **PubMed** | (((nursing home [mesh]) AND (resident[tiab] or residents[tiab])) OR (("nursing home" [tiab] OR "nursing homes" [tiab] OR "nursing facility"[tiab] OR "nursing facilities" [tiab]) AND (resident[tiab] OR residents[tiab])) OR (("long-term care facility"[tiab] OR "long-term care facilities"[tiab]) AND (resident[tiab] OR residents[tiab])) OR "home for the aged"[tiab] OR "homes for the aged"[tiab] OR "institutional* care"[tiab]) AND (Hospitalization [mesh] OR (hospitalis* [tiab] OR hospitaliz*[tiab]) OR ((hospital[tiab] OR hospitals[tiab]) AND (admit*[tiab] or admis*[tiab]or transfer[tiab])) OR (hospital[tiab] AND (treat*[tiab] OR stay[tiab] OR days[tiab] OR care[tiab]))) AND (“terminal care”[mesh] or “hospital mortality”[mesh] or “palliative care”[mesh] or “end of life” or (death or die or died or dying))  AND  (cognition OR cognitive function* OR “cognitive efficiency” OR cognitive impairment* OR memor* OR neuropsychological memor* OR alzheimer OR dementia OR forget* OR cognitive failure OR cognitive decline OR cognitive performance OR cognitive deterioration OR intelligence OR reasoning OR learning OR problem solving OR inhibition OR planning OR attention OR executive function* OR conscious* OR processing speed OR language OR “decision making” OR alertness) |
| --- | --- |
| **Scopus** | ( ( ( ( TITLE-ABS-KEY ( "nursing home" OR "nursing facility" OR "long-term care facility" ) AND TITLE-ABS ( resident ) ) OR ( TITLE-ABS-KEY ( "home for the aged" ) ) ) AND ( ( TITLE-ABS-KEY ( hospitaliz* OR hospitalis* ) ) OR ( TITLE-ABS ( hospital W/3 admit* ) ) OR ( TITLE-ABS ( hospital W/3 admis* ) ) OR ( TITLE-ABS ( hospital W/3 transfer ) ) OR ( TITLE-ABS ( hospital W/3 treat* ) ) OR ( TITLE-ABS ( hospital W/3 stay ) ) OR ( TITLE-ABS ( hospital W/3 days ) ) OR ( TITLE-ABS ( hospital W/3 care ) ) ) ) AND ( ( "end-of-life" ) OR ( death OR die OR died OR dying ) ) )  AND  ( ( cognition ) OR ( cognitive AND function* ) OR ( "cognitive efficiency" ) OR ( cognitive AND impairment* ) OR ( memor* ) OR ( neuropsychological AND memor* ) OR ( alzheimer ) OR ( dementia ) ) OR ( ( forget* ) OR ( cognitive AND failure ) OR ( cognitive AND decline ) OR ( cognitive AND performance ) OR ( cognitive AND deterioration ) OR ( intelligence ) OR ( reasoning ) ) OR ( ( learning ) OR ( problem AND solving ) OR ( inhibition ) OR ( planning ) OR ( attention ) OR ( executive AND function* ) OR ( conscious* ) ) OR ( ( processing AND speed ) OR ( language ) OR ( "decision making" ) OR ( alertness ) ) |
| **CINAHL** | ((((MH "Nursing Homes+") AND (resident OR residents)) OR ((nursing N1 (home* OR facilit*)) AND (resident OR residents)) OR ("long- term care facilit*" AND (resident OR residents)) OR "home* for the aged" OR "institutional* care") AND ((MH "Hospitalization+") OR (hospitalis* OR hospitaliz*) OR (hospital N3 (treat* OR stay OR days OR care)) OR (hospital* N3 (admit* OR admis* OR transfer)))) AND (("end of life") OR (death OR die OR died OR dying) OR ((MH "Terminal Care+")) OR ((MH "Hospital Mortality")) OR ((MH "Palliative Care")))  AND  (cognition OR cognitive function* OR “cognitive efficiency” OR cognitive impairment* OR memor* OR neuropsychological memor* OR alzheimer OR dementia OR forget* OR cognitive failure OR cognitive decline OR cognitive performance OR cognitive deterioration OR intelligence OR reasoning OR learning OR problem solving OR inhibition OR planning OR attention OR executive function* OR conscious* OR processing speed OR language OR “decision making” OR alertness) |
